# Disproportionality analysis and risk factor assessment of drug-associated thyroid dysfunction adverse events: a study based on the FAERS database

**DOI:** 10.1530/EC-25-0305

**Published:** 2025-07-17

**Authors:** Zhifang Wang, Yan Wang, Tingting Wu, Haibin Zhu, Lin Zhang, Xiaolan Liao

**Affiliations:** ^1^ School of Medicine, Tongji University, Shanghai, China; ^2^ Department of Respiratory and Critical Care Medicine, Yangpu Hospital, School of Medicine, Tongji University, Shanghai, China; ^3^ Department of Pharmacy, Yangpu Hospital, School of Medicine, Tongji University, Shanghai, China

**Keywords:** thyroid dysfunction, adverse events, FAERS database, pharmacovigilance

## Abstract

**Background:**

Thyroid dysfunction (TD) is a common adverse event (AE) associated with various pharmacological agents. However, comprehensive real-world evaluations of drug-associated TD AEs remain limited.

**Methods:**

This study utilized the U.S. FDA Adverse Event Reporting System (FAERS) database from the first quarter of 2004 to the fourth quarter of 2024 to perform analysis. The reporting odds ratio method was calculated to detect signals of TD AEs, including hyperthyroidism and hypothyroidism. Chi-square tests, Bonferroni correction, and LASSO regression were employed to select relevant predictors of TD AEs, which were subsequently analyzed using multivariate logistic regression to assess their independent associations.

**Results:**

A total of 46,725 reports with TD AEs were identified, of which 18,057 were hyperthyroidism and 28,668 were hypothyroidism. The results indicated that 21 drugs met the univariate screening criteria for hyperthyroidism, while 36 drugs met the criteria for hypothyroidism. Most of the drugs associated with TD AEs were antineoplastic agents. Multivariate analysis revealed that female patients were more likely to experience drug-associated TD AEs. In addition, 20 drugs were identified as significant factors associated with hyperthyroidism, while 34 drugs were associated with hypothyroidism.

**Conclusion:**

This study identified both known and previously unrecognized drug associations with TD AEs, particularly involving antineoplastic agents. The findings underscore the importance of routine thyroid monitoring during high-risk therapies and highlight the value of real-world pharmacovigilance in detecting emerging safety signals.

## Introduction

Thyroid dysfunction (TD), encompassing hyperthyroidism and hypothyroidism, represents a prevalent endocrine disorder associated with a substantial clinical burden. Abnormalities in thyroid function can lead to a variety of multisystem complications, including but not limited to cardiovascular disorders, neuropsychiatric disturbances, gastrointestinal dysfunction, and reproductive abnormalities (1, 2, 3, 4). Thyroid storm represents the most critical presentation of thyrotoxicosis, occurring in approximately one out of every six hospitalizations for thyrotoxicosis in the United States and carrying a 12-fold higher mortality rate (5, 6). Myxedema coma, or myxedema crisis, is the most critical and life-threatening manifestation of inadequately treated hypothyroidism (7, 8). Although rare, it is associated with high mortality and demands immediate recognition and intensive care intervention (9). Failure to manage TD appropriately may adversely affect patients’ overall quality of life.

In recent years, the increasing use of novel therapies, especially targeted agents and immunotherapies, has brought growing attention to drug-associated TD adverse events (AEs) among researchers and clinicians. Immune checkpoint inhibitors (ICIs) enhance the immune system’s ability to target cancer and have emerged as a critical advancement in contemporary oncology (10, 11). Despite their remarkable efficacy, ICIs are commonly associated with a broad spectrum of immune-related AEs (irAEs) (12, 13). Among these, TD is one of the most frequent endocrine toxicities and has garnered growing clinical attention (14, 15). Amiodarone, a commonly prescribed antiarrhythmic agent, is also well documented to be associated with adverse TD AEs (16, 17). While some studies have reported potential associations between specific drugs and TD, the current body of evidence is primarily derived from case reports, case series, or small-scale clinical studies. Comprehensive assessments using large-scale pharmacovigilance databases remain scarce.

The FDA Adverse Event Reporting System (FAERS) is a large-scale pharmacovigilance database developed and maintained by the U.S. Food and Drug Administration (FDA) (18). It operates on the spontaneous reporting system (SRS) and collects individual case safety reports (ICSRs) voluntarily submitted by healthcare professionals, patients, and pharmaceutical manufacturers (19, 20). FAERS serves as a critical resource for post-marketing drug safety surveillance, offering real-world data that complements evidence from clinical trials. By applying established data mining algorithms, researchers can identify statistical associations between drugs and AEs, enabling early detection of potential safety signals and supporting regulatory decision-making and clinical risk management (21). Accordingly, this study leverages FAERS in conjunction with data mining techniques. Through the application of disproportionality analysis, we aim to identify potential high-risk medications, detect emerging safety signals related to TD, and explore relevant risk factors. The ultimate goal is to generate evidence-based insights that support safer prescribing practices and individualized risk management in clinical settings.

## Methods

### Data sources and identification of target AEs

The AE reports data from the first quarter of 2004 (2004 Q1) to the fourth quarter of 2024 (2024 Q4) were obtained from the FAERS database. In accordance with the U.S. FDA guidelines, data preprocessing involved the removal of duplicate records before data filtering and analysis. The Medical Dictionary for Regulatory Activities (MedDRA, version 27.0) was used to identify the TD AEs. The target preferred terms were identified by applying the narrow query using Standardized MedDRA Queries (SMQs) of hyperthyroidism (SMQ code 2000161) and hypothyroidism (SMQ code 2000160).

### Data mining of drugs related to TD AEs

To evaluate the association between TD AEs and suspected drugs, a two-by-two contingency table was constructed (Table 1), and disproportionality analysis was conducted using the reporting odds ratio (ROR) with its 95% confidence interval (CI). The calculation formulas and criteria for signal detection are detailed in Table 2.

**Table 1:** Two-by-two contingency table for analysis.

Drugs	Target cases	All other AE cases	Total
Target drug	a	b	a + b
All other drugs	c	d	c + d
Total	a + c	b + d	a + b + c + d

**Table 2:** Summary of major algorithms used for signal detection.

Algorithms	Indicator	Equation	Criteria
ROR	ROR	$\;\text{ROR}\;=\;\frac{ad}{bc}$	ROR_025_ > 1, *a* ≥ 3
		${95\%\;\text{CI}\;=\;\;e}^{\ln\left(\text{ROR}\right)\;\pm \;1.96\sqrt{\frac{1}{a}+\frac{1}{b}+\frac{1}{c}+\frac{1}{d}}}$	

*a*, number of adverse event reports; CI, confidence interval; ROR, reporting odds ratio; ROR_025_, the lower limit of the 95% CI.

### Risk factors of drugs related to TD AEs analysis

The drugs associated with TD AEs, with more than 100 case reports, ROR_025_ exceeding 1, and *P-adjust* below 0.01, were included in the univariate-factor analysis. *P-adjust* was the *P*-value after the chi-square test and Bonferroni correction. LASSO regression was subsequently performed on the candidate drugs that fulfilled these screening criteria. Finally, a multifactorial logistic regression analysis of these medications was performed in conjunction with patient information (statistical significance was defined as a *P*-value less than 0.01).

### Statistical analysis

Data cleaning, AE identification, signal mining, time-to-onset analysis, and result visualization were performed using R (version 4.4.2).

## Results

### Basic characteristics of TD AE reports

**Figure 1:**
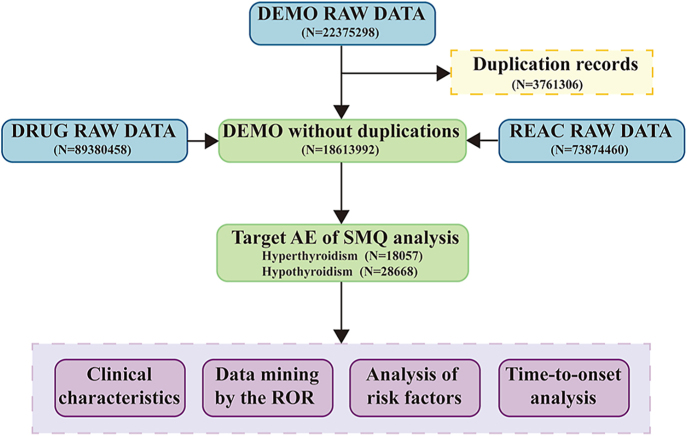
Flow chart for identification of TD reports.

The data processing flow for the entire study is shown in Fig. 1. AE reports associated with TD from the first quarter of 2004 to the fourth quarter of 2024 were extracted from the FAERS database for analysis. A total of 46,725 AE cases with abnormal thyroid function were included, of which 18,057 were hyperthyroidism and 28,668 were hypothyroidism. Table 3 demonstrates the detailed baseline information of these cases. Both groups showed a higher proportion of female patients (59.5% in hyperthyroidism and 60.9% in hypothyroidism). The median age was 56 years for hyperthyroidism and 60 years for hypothyroidism, with a high proportion of missing age data (over 30%). Median weight was slightly higher in hypothyroidism patients, but weight data were largely missing (over 66%). Most reports were submitted by physicians, followed by consumers and other health professionals, while reports from lawyers and pharmacists were less common.

**Table 3:** Demographic information of patients with thyroid dysfunction in the FAERS database.

Characteristics	Hyperthyroidism (*n* = 18,057)	Hypothyroidism (*n* = 28,668)
Gender		
Female	10,750 (59.5%)	17,462 (60.9%)
Male	5,515 (30.5%)	8,566 (29.9%)
Missing	1,792 (10.0%)	2,640 (9.2%)
Age (years)		
Median (Q1, Q3)	56 (42–68)	60 (47–70)
Missing	5,631 (31.2%)	9,670 (33.7%)
Weight (kg)		
Median (Q1, Q3)	69.0 (58.0–83.0)	70.5 (59.0–85.3)
Missing	13,219 (73.2%)	19,144 (66.8%)
Occupation of the reporter		
Consumer	5,540 (30.7%)	8,344 (29.1%)
Lawyer	325 (1.8%)	343 (1.2%)
Physician	6,051 (33.5%)	11,000 (38.4%)
Other health-professional	4,299 (23.8%)	5,986 (20.9%)
Pharmacist	765 (4.2%)	1,116 (3.9%)
Missing	1,077 (6.0%)	1,879 (6.5%)

### Drugs associated with TD AEs

**Figure 2:**
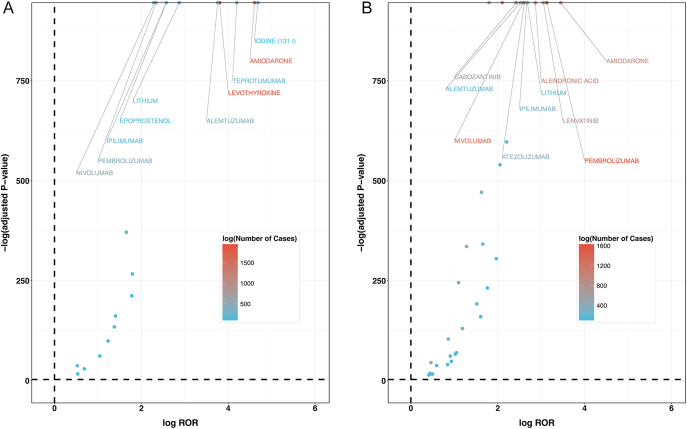
The volcano plots of drugs associated with TD. ROR, reporting odds ratio; *P-adjust*, *P*-value after Bonferroni correction. (A) Hyperthyroidism, (B) hypothyroidism.

As shown in Fig. 2, volcano plots were developed to analyze the relationship between TD AEs and the suspected drugs, which had >100 case reports, ROR_025_ > 1, and *P-adjust* <0.01. The results suggested that 21 drugs related to hyperthyroidism were eligible for screening (see Supplementary Tables S1 and S2 for details (see section on Supplementary materials given at the end of the article)), with the top five ROR values being iodine (^131^I), amiodarone, teprotumumab, levothyroxine, and alemtuzumab. In the case of hypothyroidism, 36 drugs met the screening criteria. The top five drugs with the highest ROR values were amiodarone, pembrolizumab, lenvatinib, alendronic acid, and lithium.

**Figure 3:**
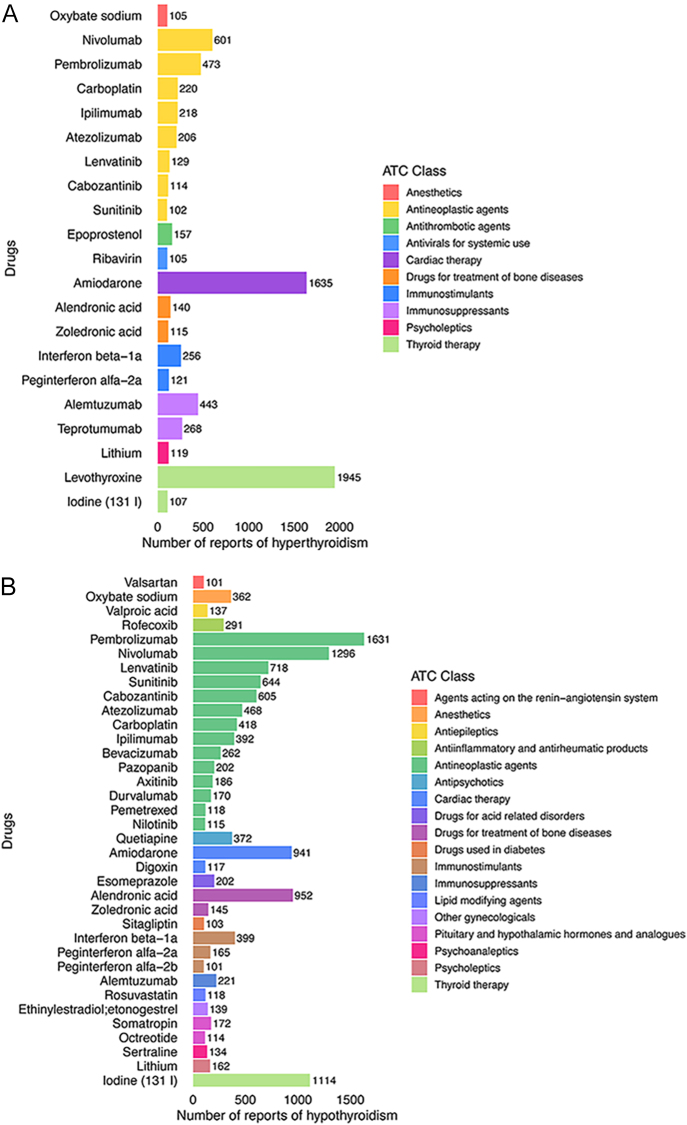
The ATC class of drugs related to TD with statistical significance (suspected drugs with >100 case reports, ROR_025_ > 1, and *P*-adjust <0.01 were extracted for single-factor analysis). (A) Hyperthyroidism, (B) hypothyroidism.

Fig. 3 shows drugs associated with TD, along with their corresponding ATC classification. For hyperthyroidism, the largest group is antineoplastic agents (8 drugs), accounting for nearly half of the total. Other categories include thyroid therapy, immunosuppressants, immunostimulants, and drugs for the treatment of bone diseases, each with two drugs. In addition, there is one drug each in cardiac therapy, psycholeptics, antithrombotic agents, antivirals for systemic use, and anesthetics. As for hypothyroidism, a total of 18 distinct ATC categories are represented among the listed drugs. The largest group is antineoplastic agents (14 drugs), followed by immunostimulants (3 drugs). Categories such as cardiac therapy, drugs for the treatment of bone diseases, and pituitary and hypothalamic hormones and analogs each include two drugs.

### Risk factors for drug-related TD AEs

**Figure 4:**
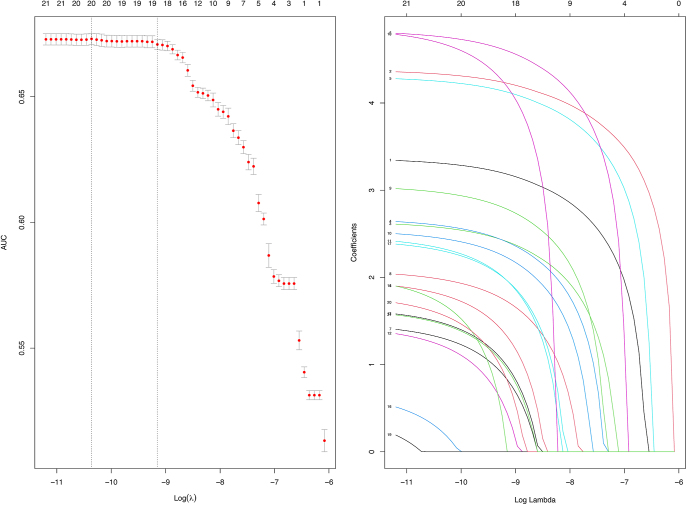
The results of the LASSO regression analysis of drugs associated with hyperthyroidism.

**Figure 5:**
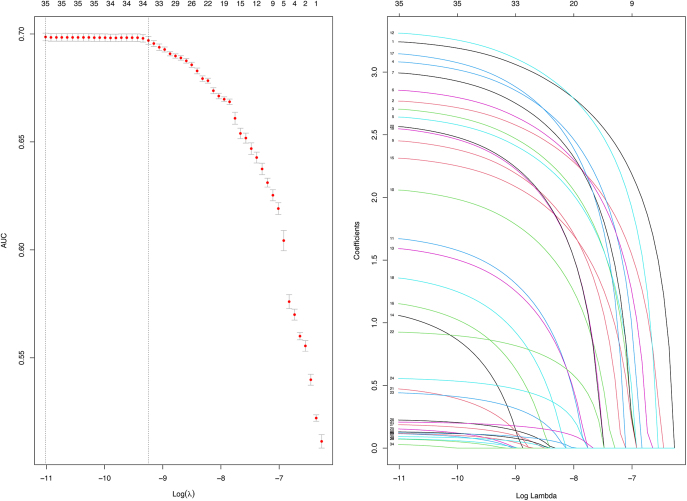
The results of the LASSO regression analysis of drugs associated with hypothyroidism.

**Figure 6:**
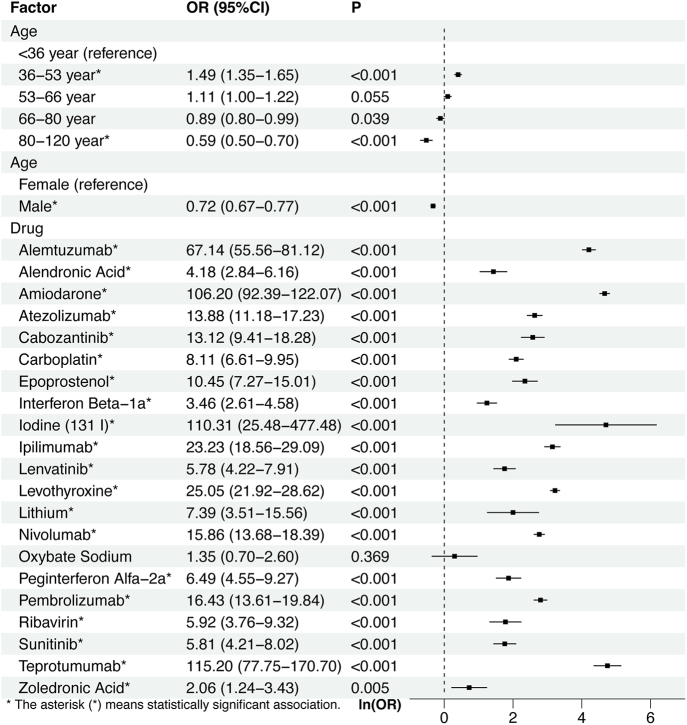
The results of the multi-factor logistic regression analysis of hyperthyroidism.

**Figure 7:**
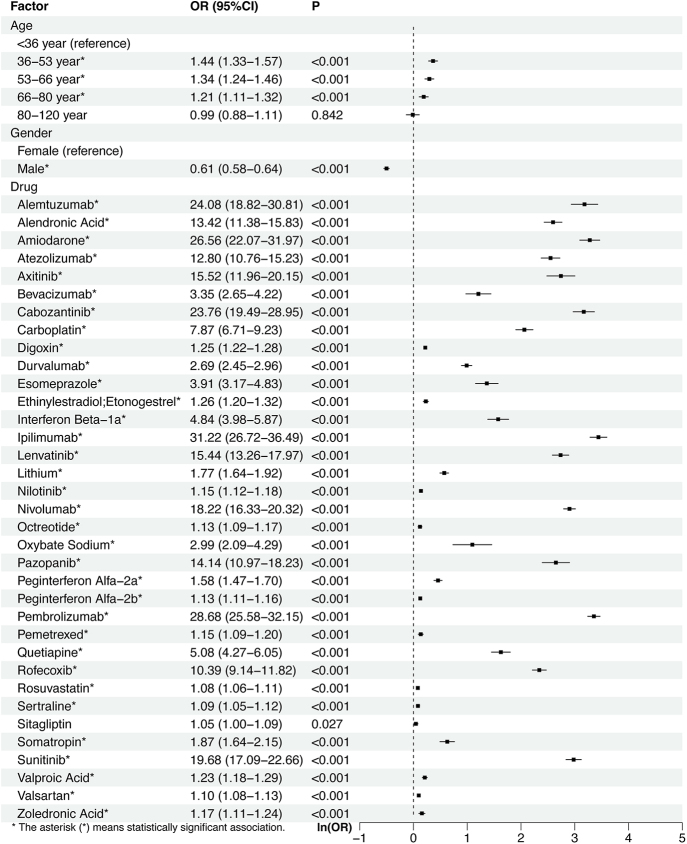
The results of the multi-factor logistic regression analysis of hypothyroidism.

A total of 21 and 36 drugs met the univariate screening criteria for hyperthyroidism and hypothyroidism, respectively. These drugs were subsequently included in the LASSO regression analysis. The results of the LASSO regression analysis are presented in Figs 4 and 5. According to the results of the LASSO regression analysis, 21 hyperthyroidism-related drugs and 35 hypothyroidism-related drugs were included in the subsequent multivariable analysis, in combination with selected patient baseline characteristics. The analysis results are presented in Figs 6 and 7.

The multivariable logistic regression analysis identified several factors significantly associated with hyperthyroidism. Among demographic variables, individuals aged 36–53 years showed an increased risk compared to those under 36 years (OR = 1.49, 95% CI: 1.35–1.65, *P* < 0.001), while older age groups (80–120 years) were associated with a decreased risk (OR = 0.59, 95% CI: 0.50–0.70, *P* < 0.001). Male patients demonstrated a significantly lower risk of hyperthyroidism compared to females (OR = 0.72, 95% CI: 0.67–0.77, *P* < 0.001). Regarding drug-related factors, 20 agents exhibited strong associations with hyperthyroidism. Notably, teprotumumab (OR = 115.20, 95% CI: 77.75–170.70, *P* < 0.001), amiodarone (OR = 106.20, 95% CI: 92.39–122.07, *P* < 0.001), iodine (^131^I) (OR = 110.31, 95% CI: 25.48–477.48, *P* < 0.001), and others were associated with markedly elevated odds.

As for hypothyroidism, compared with individuals younger than 36 years, those aged 36–53 years (OR = 1.44, 95% CI: 1.33–1.57, *P* < 0.001), 53–66 years (OR = 1.34, 95% CI: 1.24–1.46, *P* < 0.001), and 66–80 years (OR = 1.21, 95% CI: 1.11–1.32, *P* < 0.001) exhibited significantly higher odds of developing hypothyroidism. In addition, male patients had a significantly lower risk compared to females (OR = 0.61, 95% CI: 0.58–0.64, *P* < 0.001). Concerning drug-related factors, a total of 34 drugs were found to be significantly associated with hypothyroidism in the multivariate logistic regression analysis. Ipilimumab (OR = 31.22, 95% CI: 26.72–36.49, *P* < 0.001), pembrolizumab (OR = 28.68, 95% CI: 25.58–32.15, *P* < 0.001), amiodarone (OR = 26.56, 95% CI: 22.07–31.97, *P* < 0.001), and others were associated with the highest risk increases.

**Figure 8:**
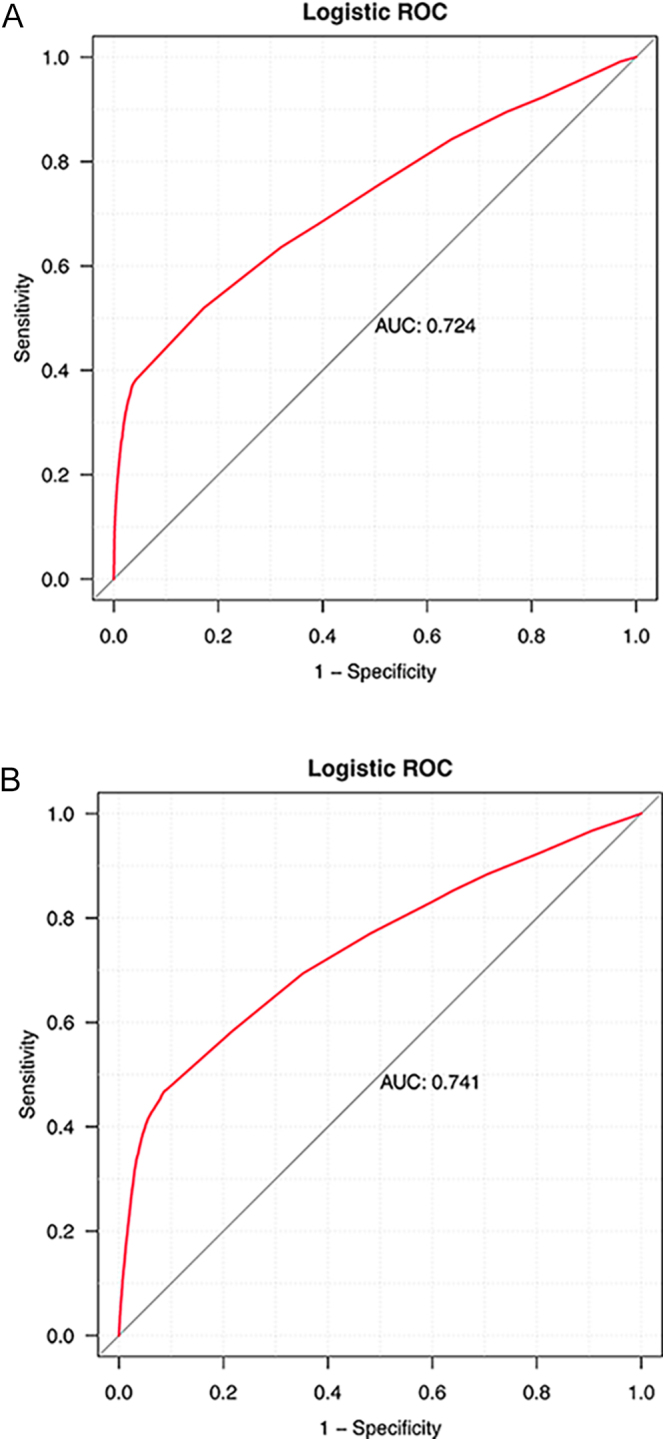
The ROC curves of drug-associated TD risk factors. ROC, receiver operating characteristic; AUC, area under the curve. (A) Hyperthyroidism, (B) hypothyroidism.

The discriminative performance of the multivariable logistic regression models for drug-associated TD was assessed using receiver operating characteristic (ROC) curves. For hyperthyroidism, the model yielded an area under the curve (AUC) of 0.724, indicating a moderate ability to distinguish between cases and non-cases (Fig. 8A). Similarly, the model for hypothyroidism demonstrated an AUC of 0.741, suggesting slightly better predictive performance (Fig. 8B). These findings indicate that the selected clinical and pharmacological factors provide acceptable predictive power for identifying patients at risk of TD.

### Time interval between drug use and the onset of TD AEs

**Figure 9:**
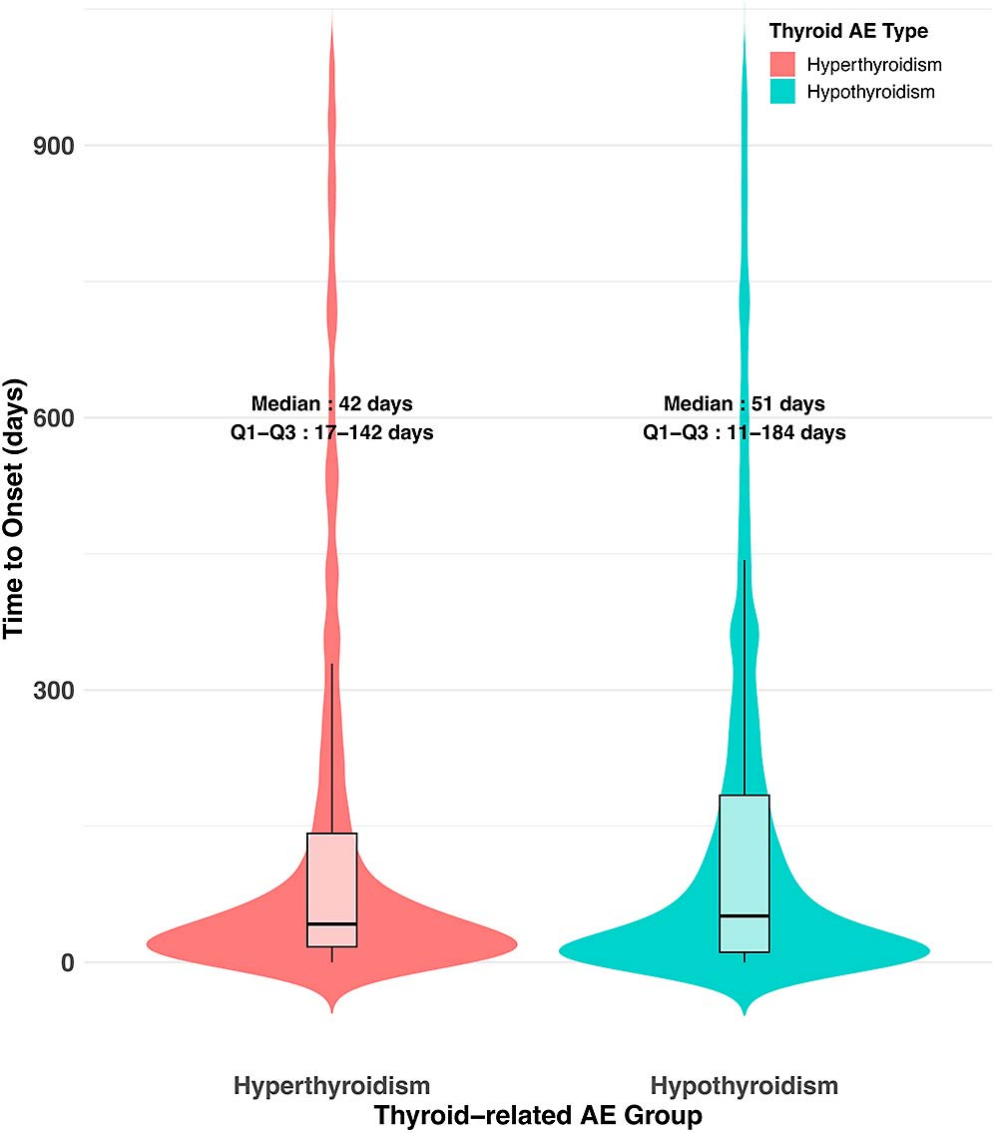
The onset time of drug-related TD.

The time-to-onset distributions of hyperthyroidism and hypothyroidism AEs were compared using violin plots (see Fig. 9). For hyperthyroidism-related AEs, the median time to onset was 42 days, with an interquartile range (IQR) of 17–142 days. In contrast, hypothyroidism-related AEs had a median time to onset of 51 days, with a broader IQR of 11–184 days. The distributions suggest that hypothyroidism tends to occur slightly later and shows greater variability in onset time compared to hyperthyroidism.

## Discussion

A comprehensive disproportionality analysis was undertaken using the FAERS database to systematically evaluate reporting signals associated with TD AEs linked to various pharmacological agents, while concurrently examining potential contributory factors that may influence their occurrence. In total, 21 drugs were identified as meeting the univariate screening criteria for hyperthyroidism, while 36 drugs met the criteria for hypothyroidism. Among the identified drugs, antineoplastic agents represented the most prominent class associated with TD AEs.

This study identified several drugs with notable reporting signals for TD AEs, including known agents such as amiodarone, lithium, and certain antineoplastic agents. Multiple ICIs were found to be associated with AE signals related to TD. Substantial variation in the incidence of TD AEs was observed across different types of ICIs (22), and the signal values identified in this study provide supporting evidence for this discrepancy. Moreover, the multivariate logistic regression analysis showed that all ICIs included in this study significantly impacted the occurrence of TD AEs. Specifically, hyperthyroidism-related AE signals were detected for ipilimumab, pembrolizumab, nivolumab, and atezolizumab. In addition, signals for hypothyroidism were observed with pembrolizumab, ipilimumab, atezolizumab, nivolumab, and durvalumab. For hyperthyroidism, ipilimumab demonstrated the strongest reporting signal, whereas for hypothyroidism, pembrolizumab exhibited the most prominent signal. These results are comparable to those reported in earlier studies. According to a meta-analysis, the incidence of TD induced by pembrolizumab, a drug of antibodies against programmed cell death-1 receptor (anti-PD-1), reached 19.8% (95% CI: 16.6–23.3%) (23). Despite earlier studies suggesting a relatively low overall incidence of TD AEs with ipilimumab (a cytotoxic T-lymphocyte–associated antigen 4 (CTLA-4) monoclonal antibody) compared to other ICIs (24, 25), this present study identified a strong reporting signal for hyperthyroidism, highlighting the need for clinical vigilance. A study indicated that autoantibodies may develop during ipilimumab treatment and could serve as potential markers of both ICI-related toxicity and therapeutic efficacy (26). Interestingly, an association has been observed between the efficacy of ICIs and the emergence of TD AEs (27, 28). The development of thyroid irAEs has been associated with the antitumor activity of ICIs, suggesting that it may serve as a surrogate marker of clinical response (29). The association between ICI-related TD AEs and therapeutic efficacy further underscores the importance of investigating the characteristics and patterns of TD AE occurrence.

ICIs exert their antitumor effects by activating the immune system; however, this immune activation is not tumor-specific and can also lead to the destruction of normal tissues and organs, which is currently considered one of the most likely mechanisms contributing to ICI-induced TD (30). Previous evidence suggests that ICIs may activate T cell-mediated pathways that disrupt TD (31, 32). Nevertheless, the precise pathophysiological mechanisms underlying ICI-related TD remain unclear and require further investigation. Some experts recommend routine monitoring of thyroid function every 4–6 weeks during ICI therapy. Mild thyrotoxicosis generally requires only careful observation, while hypothyroidism should be promptly treated with levothyroxine replacement (33). It is also advised to assess thyroid-stimulating hormone and free thyroxine (fT4) levels before initiating immunotherapy and before each of the first five treatment cycles. Thereafter, thyroid function tests should be performed at least every 3 months throughout the course of therapy. For patients with hypothyroidism, hormone replacement is the standard approach. In cases of mild thyrotoxicosis characterized by elevated heart rate but stable blood pressure, low-dose β-blockers are commonly recommended for symptomatic control (34).

Significant signals for hyperthyroidism were detected for the tyrosine kinase inhibitors (TKIs) lenvatinib, cabozantinib, and sunitinib. In addition, hypothyroidism-related signals were observed not only for these agents but also for axitinib, pazopanib, and nilotinib. These findings suggest that TD AEs may be a class effect of TKIs, likely related to their impact on vascular endothelial growth factor receptors (VEGFRs), and other signaling pathways involved in thyroid homeostasis (35, 36). A positive association between sunitinib, cabozantinib, and lenvatinib and TD AEs has been demonstrated in previous research (37). Multivariate analysis of this present study revealed that these TKIs independently contributed to the risk of TD AEs with statistical significance. Given the increasing use of these agents in oncology, clinicians should be aware of the potential for both hyperthyroid and hypothyroid states, and ensure appropriate monitoring and management throughout treatment.

Importantly, we also detected TD AEs linked to drugs not previously associated with TD AEs in product labeling. For hyperthyroidism, these included teprotumumab, lenvatinib, carboplatin, alendronic acid, zoledronic acid, and oxybate sodium. For hypothyroidism, signal-positive drugs included alendronic acid, carboplatin, pemetrexed, rofecoxib, ethinylestradiol/etonogestrel, oxybate sodium, valsartan, valproic acid, bevacizumab, sitagliptin, esomeprazole, and zoledronic acid. Nevertheless, these associations should be interpreted with caution, as they may reflect confounding by indication or demographic factors rather than true AEs. For example, teprotumumab is used to treat thyroid eye disease, the most common extrathyroidal manifestation of Graves’ disease, an autoimmune TD characterized by hyperthyroidism (38, 39, 40). Therefore, the observed association between teprotumumab and hyperthyroidism is likely due to the underlying condition rather than a direct AE of the drug. Similarly, alendronic acid is prescribed to postmenopausal women, a demographic group with a relatively high baseline prevalence of hypothyroidism, which may partially explain the observed TD signals associated with these agents (41). However, currently, there is insufficient robust clinical or epidemiological evidence to support a definitive causal relationship between bisphosphonates and TD. These findings may be attributed to the inherent limitations of the FAERS, where confounding and reporting biases can influence the detection of signals. Nevertheless, some of the newly observed TD signals associated with other drugs may still serve as valuable hypotheses for further investigation. Still, more rigorous pharmacoepidemiological studies are required to confirm or refute the potential associations observed in real-world data. It also emphasizes the need for routine thyroid function monitoring in patients undergoing such therapies, and underscores the importance of integrating real-world data into pharmacovigilance practices.

This analysis reveals that both demographic and pharmacological factors contribute to the risk of TD AEs. Male gender appeared to be a protective factor, which aligns with previous reports of female predominance in thyroid disorders, and is consistent with known epidemiological patterns (42, 43). The age-stratified analysis did not demonstrate any clear or consistent trends, suggesting that age may not serve as an independent predictor of TD AEs related to drug therapy. However, this finding may also be attributed to limitations inherent in the database, highlighting the need for further validation in prospective studies. Hyperthyroidism-related AEs presented earlier, with a median onset of 42 days, while hypothyroidism exhibited a delayed onset with a median of 51 days and a wider interquartile range. These findings are consistent with known pathophysiological mechanisms. Drug-induced hyperthyroidism often results from direct thyroid gland stimulation or thyroiditis-mediated hormone release, leading to relatively rapid symptom manifestation (44). These results underscore the necessity of prolonged and vigilant thyroid function monitoring, particularly in patients receiving therapies known to induce delayed TD. Early detection and timely intervention could mitigate potential complications and improve clinical outcomes.

It is undeniable that this study has several limitations. First, as it is based on data from the FAERS SRS, it is inherently subject to reporting bias, underreporting, inconsistencies in drug nomenclature, and potential duplication of records (45). Second, the absence of detailed clinical information, such as thyroid hormone levels, comorbidities, and treatment duration, limits the assessment of causality and clinical severity. Third, due to the observational and retrospective nature of the dataset, causal relationships between specific drugs and TD AEs cannot be definitively established (46). Finally, the relatively small number of cases in some subgroups, including age-stratified analyses, may have resulted in insufficient statistical power, potentially masking meaningful associations. Therefore, further research is needed to validate these findings in prospective studies with standardized and comprehensive clinical data.

## Conclusion

This study identified significant thyroid dysfunction adverse event (TD AE) signals associated with various drugs, particularly ICIs and TKIs, using the FAERS database. Several previously unrecognized associations between specific drugs and TD AEs were identified, highlighting the value of real-world pharmacovigilance in uncovering emerging safety signals. The findings underscore the need for routine thyroid function monitoring during high-risk therapies. Further prospective studies are warranted to validate these observations and clarify underlying mechanisms.

## Supplementary materials



## Declaration of interest

The authors declare that there is no conflict of interest that could be perceived as prejudicing the impartiality of the work reported.

## Funding

This work did not receive any specific grant from any funding agency in the public, commercial, or not-for-profit sector.

## Author contribution statement

XL and LZ led the study’s conceptualization and the design of the research protocol, establishing a strong foundation for the investigation. ZW and YW were responsible for standardizing drug nomenclature and cleaning the data to ensure the accuracy and consistency of data. XL, TW, and HZ concentrated on summarizing the data and visualizing the results. All authors played an active role in the research process, contributing to both the study’s development and the manuscript, ensuring its academic quality.

## Data availability

If appropriate, the relevant data involved in this study can be obtained from the corresponding author upon reasonable request.

## Ethical approval

The FAERS dataset is publicly accessible, and patient private information is not disclosed. Thus, ethical approval and informed consent were not required for this study.
