# Supplementary material for: Disproportionality analysis and risk factor assessment of drug-associated thyroid dysfunction adverse events: a study based on the FAERS database
Source: Endocr Connect. 2025 Jul 17;14(7):e250305. doi: 10.1530/EC-25-0305 (PMC12278363; doi:10.1530/EC-25-0305)
Supplement: Supplementary file 1 [file supplementary_materials.pdf]

Supplementary Table S1. The results of 21 drugs met the univariate screening criteria for hyperthyroidism.

Number	Drug	a	ROR (95%CI)	*P*-value	*P-adjust*
1	Iodine (131 I)	107	108.12 (88.54-132.02）	＜0.01	＜0.01
2	Amiodarone	1635	100.08 (94.93-105.51）	＜0.01	＜0.01
3	Teprotumumab	268	66.45 (58.68-75.25）	＜0.01	＜0.01
4	Levothyroxine	1945	44.79 (42.7-46.99）	＜0.01	＜0.01
5	Alemtuzumab	443	42.81 (38.88-47.13）	＜0.01	＜0.01
6	Lithium	119	17.69 (14.75-21.22）	＜0.01	＜0.01
7	Epoprostenol	157	13.11 (11.19-15.35）	＜0.01	＜0.01
8	Ipilimumab	218	13.1 (11.45-14.98）	＜0.01	＜0.01
9	Pembrolizumab	473	10.39 (9.48-11.39）	＜0.01	＜0.01
10	Nivolumab	601	9.96 (9.18-10.81）	＜0.01	＜0.01
11	Atezolizumab	206	9.8 (8.54-11.25）	＜0.01	＜0.01
12	Lenvatinib	129	6.02 (5.06-7.16）	＜0.01	＜0.01
13	Ribavirin	105	5.92 (4.88-7.17）	＜0.01	＜0.01
14	Carboplatin	220	5.23 (4.58-5.98）	＜0.01	＜0.01
15	Alendronic Acid	140	4.07 (3.45-4.81）	＜0.01	＜0.01
16	Peginterferon Alfa-2A	121	3.97 (3.32-4.75）	＜0.01	＜0.01
17	Cabozantinib	114	3.44 (2.86-4.14）	＜0.01	＜0.01
18	Sunitinib	102	2.84 (2.34-3.46）	＜0.01	＜0.01
19	Zoledronic Acid	115	1.99 (1.65-2.39）	＜0.01	＜0.01
20	Oxybate Sodium	105	1.71 (1.41-2.07）	＜0.01	＜0.01
21	Interferon Beta-1A	256	1.69 (1.5-1.91）	＜0.01	＜0.01

a, number of adverse event reports; ROR, reporting odds ratio; CI, confidence interval.

Supplementary Table S2. The results of 36 drugs met the univariate screening criteria for hypothyroidism.

Number	Drug	a	ROR (95%CI)	*P*-value	*P*-adjust
1	Amiodarone	941	31.51 (29.48-33.67）	＜0.01	＜0.01
2	Pembrolizumab	1631	22.86 (21.72-24.04）	＜0.01	＜0.01
3	Lenvatinib	718	21.17 (19.64-22.83）	＜0.01	＜0.01
4	Alendronic Acid	952	17.56 (16.45-18.74）	＜0.01	＜0.01
5	Lithium	162	14.63 (12.51-17.1）	＜0.01	＜0.01
6	Ipilimumab	392	14.39 (13.01-15.92）	＜0.01	＜0.01
7	Atezolizumab	468	13.68 (12.47-15）	＜0.01	＜0.01
8	Nivolumab	1296	13.27 (12.54-14.03）	＜0.01	＜0.01
9	Alemtuzumab	221	12.43 (10.87-14.21）	＜0.01	＜0.01
10	Cabozantinib	605	11.36 (10.47-12.32）	＜0.01	＜0.01
11	Sunitinib	644	11.19 (10.34-12.1）	＜0.01	＜0.01

Number	Drug	a	ROR (95%CI)	*P*-value	*P*-adjust
12	Durvalumab	170	9.07 (7.79-10.56）	＜0.01	＜0.01
13	Iodine (131 I)	1114	8.19 (7.64-8.78）	＜0.01	＜0.01
14	Axitinib	186	7.79 (6.74-9.01）	＜0.01	＜0.01
15	Digoxin	117	7.16 (5.97-8.6）	＜0.01	＜0.01
16	Carboplatin	418	6.05 (5.49-6.67）	＜0.01	＜0.01
17	Pemetrexed	118	5.81 (4.84-6.96）	＜0.01	＜0.01
18	Pazopanib	202	5.24 (4.56-6.02）	＜0.01	＜0.01
19	Rofecoxib	291	5.09 (4.54-5.72）	＜0.01	＜0.01
20	Peginterferon Alfa-2B	101	4.99 (4.1-6.07）	＜0.01	＜0.01
21	Ethinylestradiol; Etonogestrel	139	4.56 (3.86-5.4）	＜0.01	＜0.01
22	Oxybate Sodium	362	3.6 (3.25-4）	＜0.01	＜0.01
23	Peginterferon Alfa-2A	165	3.28 (2.81-3.82）	＜0.01	＜0.01
24	Quetiapine	372	3 (2.71-3.32）	＜0.01	＜0.01
25	Octreotide	114	2.86 (2.38-3.44）	＜0.01	＜0.01
26	Nilotinib	115	2.78 (2.31-3.34）	＜0.01	＜0.01
27	Valsartan	101	2.54 (2.09-3.09）	＜0.01	＜0.01
28	Valproic Acid	137	2.48 (2.1-2.93）	＜0.01	＜0.01
29	Bevacizumab	262	2.37 (2.1-2.68）	＜0.01	＜0.01
30	Sitagliptin	103	2.33 (1.92-2.83）	＜0.01	＜0.01
31	Esomeprazole	202	1.81 (1.57-2.08）	＜0.01	＜0.01
32	Rosuvastatin	118	1.65 (1.38-1.98）	＜0.01	＜0.01
33	Sertraline	134	1.6 (1.35-1.9）	＜0.01	＜0.01
34	Interferon Beta-1A	399	1.59 (1.44-1.76）	＜0.01	＜0.01
35	Somatropin	172	1.55 (1.33-1.8）	＜0.01	＜0.01
36	Zoledronic Acid	145	1.51 (1.29-1.78）	＜0.01	＜0.01

a, number of adverse event reports; ROR, reporting odds ratio; CI, confidence interval.
